# Gastrointestinal parasite diversity of South American camelids (Artiodactyla: Camelidae): First review throughout the native range of distribution

**DOI:** 10.1016/j.ijppaw.2022.10.001

**Published:** 2022-10-26

**Authors:** Victoria Cañal, María Ornela Beltrame

**Affiliations:** Grupo de investigación, Paleoparasitología. Instituto de Investigaciones en Producción, Sanidad y Ambiente (IIPROSAM), Facultad de Ciencias Exactas y Naturales, UNMdP-CONICET, Consejo Nacional de Investigaciones Científicas y Técnicas (CONICET), Juan B. Justo 2250, CP 7600, Mar del Plata, Buenos Aires, Argentina

**Keywords:** *Lama*, *Vicugna*, Alpacas, Llamas, Vicuñas, Guanacos, Endoparasites

## Abstract

In South America inhabit an endemic group of ungulates adapted to extreme environments: the South American camelids (SAC), a key component of the Andean biocultural heritage. Until today, SAC are the most important factor of Andean economies and social and ritual life. SAC include two wild species, the guanaco (*Lama guanicoe)* and the vicuña (*Vicugna vicugna)*, and two domestic species, the llama (*Lama glama)* and the alpaca (*Vicugna pacos).* Endoparasitosis are one of the most common diseases in SAC, and have great economic and health relevance. Despite this, there is a lack of knowledge on this concern. The main objective of this work was to conduct the first systematic review of the diversity of gastrointestinal parasites of SAC throughout the entire native range of distribution and to identify several gaps in knowledge. The PRISMA protocol was performed and a total of 101 documents were summarized. At least 36 parasitic helminths and five *Eimeria* spp. were registered. This work highlights the need for a greater number of works to know with more certainty the parasitic fauna of camelids in the past and present, in order to achieve predictions that allow proper management of camelids for their future conservation. Furthermore, concerted research efforts are needed to understand the biology, epidemiology, diagnosis and distribution of the parasitosis of SAC along the entire distribution range to guide conservation decisions.

## Highlights

• Endoparasitosis are one of the most common diseases in SAC.
• First review of parasites diversity of SAC throughout its total range of distribution.
• At least 36 parasitic helminths and 5 *Eimeria* spp. were registered in camelids.
• The registered parasites are mostly generalist parasites.
• A better understanding of the extent and impact of parasites of SAC is needed.

## Graphical abstract

**Figure:**
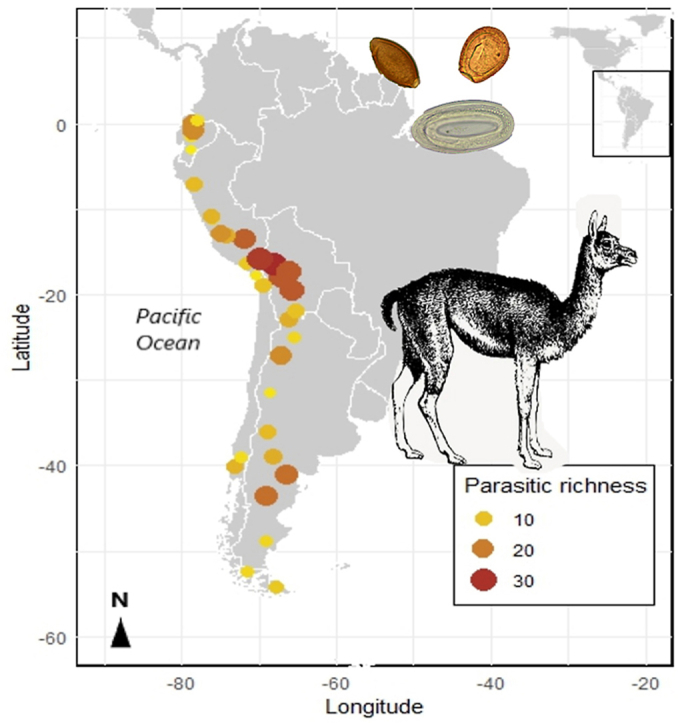

## 1 Introduction

The South American camelids (SAC) (Artiodactyla, Camelidae) are a key component of the Andean biocultural heritage (Vilá and Arzamendia, 2020) and have occupied a central role in the development of Andean societies, both for ancient hunter-gatherers and for more recent pastoralists and farmers. SAC were the most important factor in Andean economies and social and ritual life throughout time. SAC include two wild species, the guanaco (*Lama guanicoe)* and the vicuña (*Vicugna vicugna)*; and two domestic species, the llama (*Lama glama)* and the alpaca (*Vicugna pacos)* (Wheeler et al., 2006; Yacobaccio, 2021). This is an endemic group of ungulates adapted to extreme environments with a wide distributionin in arid and semiarid ecosystems from Argentina, Bolivia, Chile, Ecuador and Peru, mainly from 3000 to 5000 m.a.s.l. (Franklin, 2011). The original distribution of SAC includes the Andean high-altitude grasslands, the Altiplano and the Patagonian arid steppes (Vilá and Arzamendia, 2020). The distribution of guanacos includes a wide diversity of open habitats and temperate forest environments of Peru, Bolivia, Chile and Argentina, including Patagonian steppes. The distribution of vicuñas is limited to Northern Argentina, Chile, Peru and Bolivia, restricted to high-altitude Puna environments, above 3400 m.a.s.l. (Vilá, 2012). In pre-Hispanic times, llamas inhabit the Andean regions of Peru, Bolivia, Argentina and Chile and the alpacas were restricted to high and humid environments from the Puna of Peru, Bolivia and Chile (Yacobaccio, 2021). Under the dominion of the Incas (1470–1532), the llama distribution reached the southern Colombia and central Chile. There is no evidence of the presence of alpacas in pre-Columbian sites from Argentina (Olivera and Grant, 2009) and Ecuador (Miller and Gill, 1990) being introduced in these regions later.

Today, husbandry of SAC is an important socioeconomic activity for the Andean populations of South America. Recently, the breeding of domestic camelids also began to have great interest in other parts of the world. Numerous publications have been reported the relevance of parasites of SAC. Endoparasitosis are one of the most common diseases of SAC and have great economic and health relevance. Host-specific parasites and generalistic parasites shared with domestic ruminants such as sheep and goats are well known and have been widely described in the literature (e.g. Navone and Merino 1989; Leguia, 1991; Beldomenico et al., 2003; Aguirre and Cafrune, 2007; Arias-Pacheco et al., 2021). It is known that camelids are parasitized by gastrointestinal nematodes, trematodes and cestodes, and by coccidians, among other parasites. Many of them can cause serious diseases (Fowler, 2010) and can be transmitted to humans, including hydatidosis, fascioliasis, sarcocystosis and toxoplasmosis. The knowledgement of the diversity, spread, and evolution of parasites of SAC play a very important role in the understanding of the behavioral ecology, health, and camelids conservation. Despite this, there is a lack of knowledge about a global vision of gastrointestinal parasite diversity throughout the entire distribution range. The main objective of this work was to conduct the first systematic review of the diversity of gastrointestinal parasites of SAC throughout the native range of distribution and to identify several gaps in knowledge.

## 2 Materials and methods

The research was conducted using the systematic approach of the Preferred Reporting Items for Systematic Review and Meta-Analyses (PRISMA) protocol guidelines (Shamseer et al., 2015).

### 2.1 Selection criteria

The literature used in this review included publications reporting on gastrointestinal parasites of SAC. The following list gives the criteria used in the selection of publications.

The inclusion criteria were:

• Scientific peer-reviewed, scientific papers, conference proceedings and theses (PhD and MSc Thesis and Final Degree Projects) were included.
• Literature published in English and Spanish-written in order to include research with local, regional and global impact.
• Gastrointestinal parasite analysis of SAC in the natural range of distribution in order to conduct the first review in the subject and to identify gaps in knowledge.

The exclusion criteria were:

• Research papers conducted in SAC from sites outside the natural range of distribution.
• Research papers on topics other than gastrointestinal parasites of SAC.

### 2.2 Search strategy and data

#### 2.2.1 Identification

**Fig. 1:**
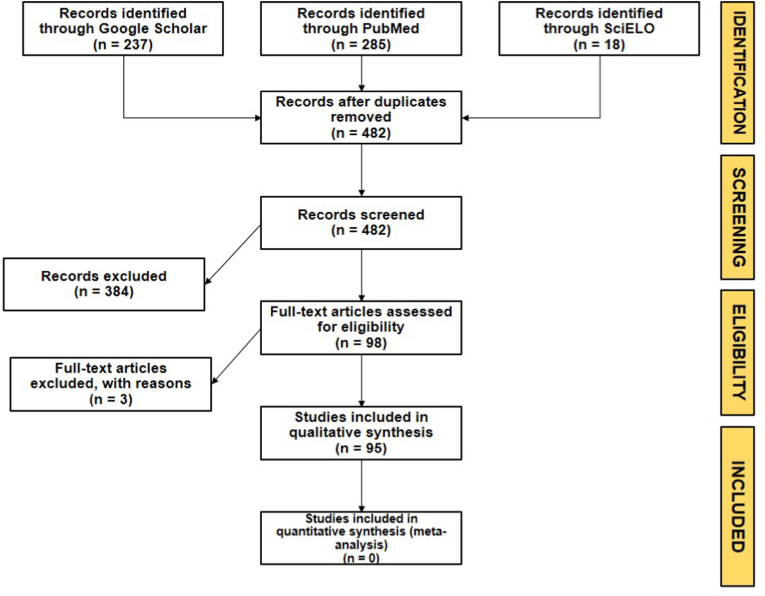
PRISMA flowchart of the systematic review process.

The study was focused on gastrointestinal helminths and *Eimeria* spp., hereafter “parasites”. The literature research was carried out on internet through the Google Scholar platform (https://scholar.google.com), the PubMed platform (https://pubmed.ncbi.nlm.nih.gov), and the SciELO platform (https://scielo.org/es/). The following keywords were used for the research: “endoparasites”, “gastrointestinal”, “intestinal”, “parasites”, “helminths”, “camelids”, “South American Camelids”, “SAC”, “endoparásitos”, “parásitos”, “intestinales”, gastrointestinales”, “helmintos”, “camélidos”, “camélidos sudamericanos”, “CSA”, “guanaco”, “*Lama*”, “*Vicugna*”, “*guanicoe*”, “*pacos*”, “*glama*”, “llama”, “alpaca”, “vicuña”, “*Eimeria*”, “Coccidia”. The search rule used in English was (endoparasites OR gastrointestinal OR intestinal) AND (parasites OR helminths OR *Eimeria* OR coccidia) AND (camelids OR South American camelids OR SAC OR guanaco OR vicuña OR llama OR alpaca OR *Lama* OR *Vicugna*) AND (*guanicoe* OR *glama* OR *vicugna* OR *pacos*). The search rule used in Spanish was (endoparásitos OR parásitos OR helmintos OR *Eimeria*) AND (intestinales OR gastrointestinales) AND (camélidos OR camélidos sudamericanos OR CSA OR guanaco OR vicuña OR llama OR alpaca OR *Lama* OR *Vicugna*) AND (*guanicoe* OR *glama* OR *vicugna* OR *pacos*). The search was conducted in titles, abstracts and keywords in the above-cited databases, following the selection criteria. The snowball effect in the reference lists was used to increase the scope of the search. The initial search process generated 3960 academic papers from Google Scholar, and additional 285 papers from PubMed and 18 papers from SciELO. The publication retrieval from Google Scholar was scaled down to 237 after removing all parasite papers that did not represent the objective of this review. A flowchart of the PRISMA phases of the search is presented in Fig. 1.

#### 2.2.2 Screening

After the initial search and paper retrieval, 540 academic papers were collected. After removing duplicate information, 482 publications remained. Subsequently, the generated papers were screened by applying the inclusion and exclusion criteria. A total of 98 academic papers were included for quality assessment.

#### 2.2.3 Eligibility

The studies identified after applying the inclusion and exclusion criteria underwent further evaluation to ensure the quality of the research articles. The theses that contained only information published in scientific journals were eliminated. In total, 3 theses were excluded.

#### 2.2.4 Included papers

A total of 95 publications were included in this review. From all reviewed documents, were extracted data regarding geographic location (country and region), number of samples evaluated, number of positive samples, taxa and prevalence of parasites reported, remarks (type of sample, animal characteristics, study remarks) and type of publication, being the data extraction performed by one author with verification by another, as the PRISMA protocol suggest (Shamseer et al., 2015).

## 3 Results

The present review includes documents from the period between 1963 and 2022. The information was retrieved from 95 publications and 6 more citations were added (there was no access to the original work), which makes a final number of 101 publications. A total of 74 scientific researchs, 27 theses (PhD and MSc Thesis and Final Degree Projects), four abstracts of scientific meetings and one FAO project were recopiled. The name of parasites was included exactly as reported in the retrieved publications.

**Fig. 2:**
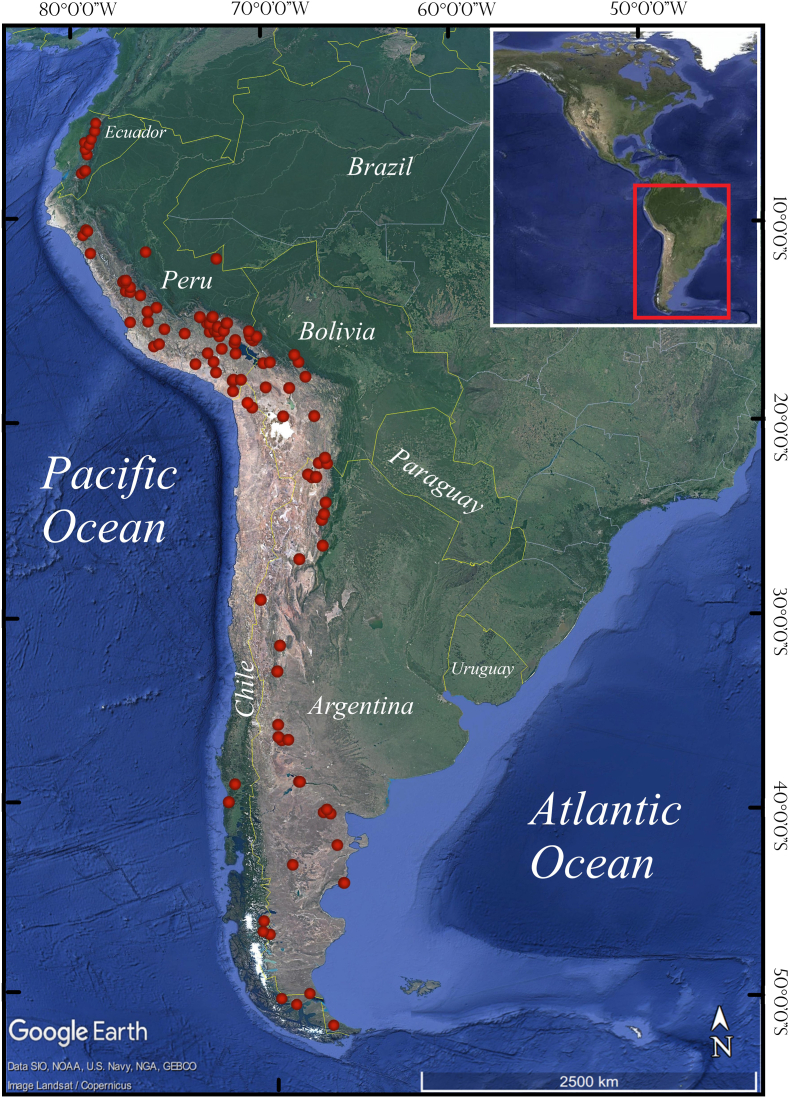
Geographical location of the documents compiled in the present review (red dots). (For interpretation of the references to colour in this figure legend, the reader is referred to the Web version of this article.)

The documents summarized belong to five countries (Argentina, Bolivia, Chile, Peru and Ecuador), with the alpaca being the most studied species of SAC, (49.5% of the total documents), followed by the guanaco (23.8%) and finally the llama and vicuña (both 18.8%). The geographical location of the documents summarized is shown in Fig. 2. The map was elaborated with the Google Earth platform.

**Fig. 3:**
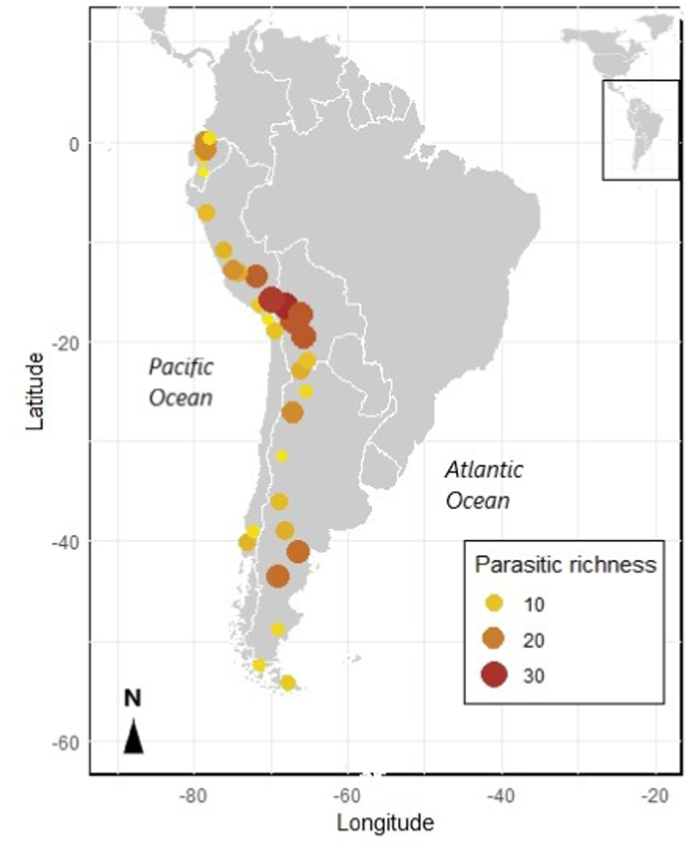
Parasitic richness of South American camelid throught the native distribution range based on data available to date.

The reports of parasites of alpacas are summarized in Table 1. The 80% of the documents refer to alpacas from Peru, 12% from Ecuador, 4% from Chile and 4% from Bolivia. The reports of parasites of llamas are summarized in Table 2. The 47.4% of the recopiled documents belong to Argentina, 21% to Peru, 15.8% to Chile, 10.5% to Ecuador and 5.3% belong to Bolivia. The Table 3 summarized the recopiled documents of parasites of vicuñas. The 42.1% of the documents refer to vicuñas from Peru, 31.6% from Argentina and 21% from Bolivia and only one document refers to vicuñas from Ecuador (5.3%). The reports of parasites of guanacos are summarized in Table 4. Most of the recopiled documents belong to Argentina (83.33%), while documents from Chile and Peru represent both 8.33%. The data collected throught the entire native range of SAC distribution displayed that the highest species richness of gastrointestinal parasites are found in southern Peru, western Bolivia and central Patagonia. It is important to highlight that this data was elaborated from the information available to date. The parasitic richness found in SAC compiled from the information extracted is represented in Fig. 3.

**Table 1:** Gastrointestinal helminths and *Eimeria* spp. reported in alpacas (*Lama pacos*) across the entire nature distribution range. (* Calculated with published data, NR: no reported).

Country	Region	No. tested samples	No. positive (%)	Reported parasites (%)	Remarks	Type of publication	Reference
Chile	Valdivia	47	NR	*Nematodirus spathiger*	Rectal samples	Scientific research	Valenzuela et al. (1998)
				*Nematodirus filicolis*			
				*Ostertagia* sp*.*			
				*Trichostrongylus* sp*.*			
				*Cooperia* sp*.*			
				Strongylida			
				*Trichuris* sp*.*			
				*Capillaria* sp*.*			
	Arica-Parinacota	494	52 (10.53)	*Fasciola hepatica*	Rectal samples Only *Fasciola* study	Scientific meeting	Zamorano et al. (2012)
Bolivia	La Paz	22	(59.1)	*Fasciola hepatica*	Only abstract access	Scientific research	Ueno et al. (1975)
	La Paz	55	54 (98.0)	Eimeria punoensis (67.37)*	Fecal samples	Scientific research	Beltrán-Saavedra et al. (2014)
				Eimeria alpacae (16.36)*			
				Eimeria macusaniensis (12.72)*			
				*Marshallagia* sp. (47.27)*			
				*Lamanema* spp. (5.45)*			
				*Nematodirus* spp. (69.09)*			
				Strongylida (52.72)*			
				*Capillaria* sp. (25.45)*			
				*Trichuris* sp. (36.36)*			
				Moniezia expansa (5.45)*			
				Moniezia benedeni (10.90)*			
				*Fasciola* sp. (1.81)*			
Peru	NR	NR	NR	*Lamanema chavezi*	Only abstract access	Scientific research	Becklund (1963)
				*Nematodirus lamae*			
	Cuzco and Puno	12	NR	*Eimeria lamae*	Rectal samples	Scientific research	Guerrero (1967)
				*Eimeria alpacae*	First description		
				*Eimeria punoensis*	Only *Eimeria* study		
	Puno	NR	NR	*Haemonchus* sp.	No access to original document	Scientific research	Guerrero Diaz (1970) (in Navone and Merino, 1989)
				*Ostertagia* sp.			
				*Trichostrongylus* sp.			
				*Cooperia* sp.			
				*Nematodirus* sp.			
				*Oesophagostomum* sp.			
				*Chabertia* sp.			
				*Trichuris* sp.			
				*Graphinema* sp.			
				*Lamanema* sp.			
				*Mazamastrongylus (Spiculopteragia) sp.*			
				*Camelostrongylus* sp.			
				*Dictyocaulus* sp.			
				*Fasciola* sp.			
				*Moniezia* sp.			
				*Eimeria* sp.			
	Puno	NR	NR	*Eimeria lamae*	Fecal samples	Scientific research	Guerrero et al. (1971)
				*Eimeria alpacae*	First report *E. macusaniensis*		
				*Eimeria punoensis*	Only *Eimeria* study		
				*Eimeria macusaniensis*			
	Junín	NR	NR	*Eimeria ivitaensis*	Rectal samples	Scientific research	Leguía and Casas (1998)
					First description		
					Only *Eimeria* study		
	Junín	280	NR	*Fasciola hepatica (7.1)*	Fecal samples	Scientific research	Neyra et al. (2002)
				*Trichuris* sp*. (40.0)*			
				*Nematodirus* sp*. (34.6)*			
				*Lamanema* sp*. (12.8)*			
				*Eimeria* sp*. (11.8)*			
	Cuzco	7	NR	*Eimeria lamae*	Necropsy	Scientific research	Palacios et al. (2004)
				*Eimeria macusaniensis*	Dead animals with clinical signs of diarrhea		
				*Eimeria ivitaensis*	Only *Eimeria* study		
	Cuzco	48	11 (23.0)	*Eimeria macusaniensis*	Intestinal samples	Scientific research	Palacios et al. (2005)
				*Eimeria lamae*	Dead animals with diarrhea		
				*Eimeria punoensis*	Only *Eimeria* study		
				*Eimeria alpacae*			
	NR	40	12	*Fasciola hepatica*	Fecal samples	Scientific research	Li et al. (2005)
					ELISA method		
					Only *Fasciola* study		
	Cuzco	NR	NR	*Eimeria macusaniensis*	Histopathological examination	Scientific research	Palacios et al. (2006)
				*Eimeria ivitaensis*	Only *Eimeria* study		
	Ayacucho	10	(100)	*Fasciola hepatica*	Fecal and blood samples	Scientific research	Ciprián (2007)
					Necropsy		
					Only *Fasciola* study		
	Puno	NR	(3.03)	Coccidia *(25.44)*	Necropsy	Scientific research	Paredes et al. (2009)
				Vermes *(10.52)*			
				*Dictyocaulus* sp. *(0.88)*			
	Puno and Cuzco	108	33 (30.55)	*Eimeria macusaniensis*	Intestinal samples	Scientific research	Rosadio et al. (2010)
	South of Peru	316	NR	*Eimeria macusaniensis (56.5)*	Only *Eimeria* study	Scientific research	Cordero et al. (2011) (in Dubey, 2018)
	Puno	60	NR	*Strongylus* sp.	Rectal samples	Scientific research	Marino (2011)
				*Nematodirus* sp.			
				*Nematodirus lamae*			
				*Lamanema chavezi*			
	Huancavelica	161	NR	*Eimeria* spp. *(31.37)*	Fecal samples	Scientific research	Rosadio et al. (2012)
				*Eimeria macusaniensis (4.3)*	Adults		
	Puno	478	418 (87.5)	*Eimeria lamae (60.4)*	Fecal samples	Scientific research	Rodríguez et al. (2012)
				*Eimeria alpacae (45.6)*	Only *Eimeria* study		
				*Eimeria punoensis (30.0)*			
				*Eimeria macusaniensis (50.4)*			
				*Eimeria ivitaensis (6.24)*			
	Cuzco	30	NR	*Eimeria lamae*	Fecal samples	Thesis	Mamani (2012)
				*Eimeria alpacae*			
				*Eimeria punoensis*			
				*Eimeria macusaniensis*			
				*Eimeria ivitaensis*			
				*Nematodirus spathiger*			
				*Nematodirus lamae*			
				*Lamanema chavezi*			
				*Trichuris* spp*.*			
				*Capillaria* spp*.*			
				Strongylida			
	Huancavelica	366	(59.02)	*Eimeria* spp*.*	Rectal samples	Thesis	Auris Bellido and Santiago Cahuana (2013)
					Young animals with diarrhea		
	Cuzco	1001	Helminthes (68.4)	*Nematodirus (54.0)*	Rectal samples	Scientific research	Pérez et al. (2014)
			*Eimeria* spp. (61.5)	Strongylida *(16.3)*			
				*Trichuris (17.5)*			
				*Capillaria (5.1)*			
				*Lamanema (4.5)*			
				*Moniezia (6.3)*			
				*Eimeria alpacae (42.0)*			
				*Eimeria punoensis (31.0)*			
				*Eimeria lamae (20.0)*			
				*Eimeria macusaniensis (7.0)*			
				*Cooperia (40.0)*			
				*Ostertagia (22.0)*			
				*Trichostrongylus (20.0)*			
				*Oesophagostomum (16.0)*			
				*Bunostomum (2.0)*			
	Junín, Jauja	103	(73.8)	*Fasciola hepatica*	Rectal samples	Scientific research	Flores et al. (2014)
					Only *Fasciola* study		
	Puno	1319	(63.9)	*Nematodirus* spp. *(52.8)*	Rectal samples	Scientific research	Contreras et al. (2014)
				Strongylida *(4.9)*			
				*Trichuris* spp. *(10.8)*			
				*Capillaria* spp. *(1.8)*			
				*Lamanema* spp. *(0.7)*			
				*Moniezia* spp. *(9.6)*			
				*Cooperia* spp. *(37.0)*			
				*Oesophagostomum* spp. *(23.0)*			
				*Trichostrongylus* spp. *(20.0)*			
				*Ostertagia* spp. *(14.0)*			
				*Bunostomum* spp. *(3.0)*			
				*Haemonchus* spp. *(3.0)*			
	Cajamarca	10	9 (90.0)	*Nematodirus* sp*. (70.0)*	Necropsy	Thesis	Roncal Narváez (2014)
				*Bunostomum* sp*. (50.0)*	Sacrificed alpacas from slaughterhouse		
				*Trichuris* sp*. (40.0)*			
				*Moniezia* sp*. (30.0)*			
				*Ostertagia* sp*. (30.0)*			
				*Trichostrongylus* sp*. (20.0)*			
	Cajamarca	151	20 (13.25)	*Fasciola hepatica*	Rectal samples	Thesis	López Mejía (2014)
					Only *Fasciola* study		
	Puno	369	(54.20)	*Nematodirus lamae*	Rectal samples	Thesis	Farfán (2014)
				*Lamanema chavezi*			
				*Trichostrongylus*			
				*Trichuris* sp.			
				*Moniezia benedeni*			
				*Moniezia expansa*			
	Puno	20	NR	*Lamanema chavezi*	Rectal samples and intestinal segments	Scientific research	Angulo et al. (2015)
					Redescription		
					Only *Lamanema* study		
	Puno	51	14 (27.5)	*Eimeria* spp.	Necropsy	Abstract of Scientific meeting	Díaz et al. (2015)
					Intestinal samples		
	Puno	30	NR	*Eimeria punoensis*	Rectal samples and necropsy	Thesis	Quina Quina (2015)
				*Eimeria lamae*			
				*Eimeria macusaniensis*			
				*Eimeria alpacae*			
				*Eimeria ivitaensis*			
				*Strongylus* sp.			
				*Nematodirus* spp.			
				*Nematodirus spathiger*			
				*Nematodirus lamae*			
				*Trichuris* sp.			
				*Lamanema chavezi*			
				*Capillaria* sp.			
				*Moniezia benedeni*			
				*Moniezia expansa*			
	Puno	350	224 (64.3)	*Eimeria lamae*	Fecal samples from unweaned alpacas	Scientific research	Díaz et al. (2016)
				*Eimeria alpacae*	Only *Eimeria* study		
				*Eimeria punoensis*			
				*Eimeria macusaniensis*			
				*Eimeria ivitaensis*			
	Pasco	160	NR	Strongylida *(28.1)*	Rectal samples	Scientific research	Masson et al. (2016)
				*Eimeria ivitaensis (6.9)*			
				*Eimeria macusaniensis (41.9)*			
				*Nematodirus* spp*. (26.3)*			
				*Trichuris* sp*. (20.0)*			
				*Capillaria* sp*. (5.0)*			
				*Lamanema chavezi (3.8)*			
				*Cooperia* spp*.*			
				*Oesophagostomum* spp*.*			
				*Teladorsagia circumcincta*			
				*Ostertagia ostertagi*			
				*Trichostrongylus* spp*.*			
	Pasco and Junín	60	(73.3)	*Eimeria* spp*. (43.3)*	Rectal samples and necropsy	Scientific research	Lucas et al. (2016)
				*Eimeria alpacae*	Dead calves with diarrhea		
				*Eimeria macusaniensis*			
				*Eimeria lamae*			
				*Nematodirus* sp*. (40.0)*			
				Strongylida *(18.3)*			
				*Trichuris* sp*. (1.6)*			
	Puno	1319	(52.4)	*Eimeria alpacae (31.5)*	Rectal samples	Scientific research	Camareno et al. (2016)
				*Eimeria lamae (2.3)*	Only *Eimeria* study		
				*Eimeria punoensis (66.2)*			
				*Eimeria macusaniensis (8.7)*			
				*Eimeria ivitaensis (0.7)*			
	Huancavelica	190	(81.88)	*Eimeria macusaniensis*	Rectal samples	Thesis	Lizana Hilario (2016)
	Puno	45	NR	*Eimeria* spp*.*	Intestinal sample	Thesis	Chirinos (2017)
	Arequipa, Tacna	346	(69.65)	*Nematodirus* spp*. (46.53)*	Rectal samples	Thesis	Torres Huacani (2017)
				*Trichuris* spp*. (15.61)*			
				*Capillaria* spp*. (13.01)*			
				*Lamanema chavezi (1.45)*			
				Strongylida *(4.34)*			
				*Moniezia expansa (6.65)*			
				*Eimeria* spp*. (45.66)*			
	Pasco	238	51 (21.43)	*Nematodirus* sp*.*	Rectal samples	Thesis	Puicón (2018)
		178	7 (3.93)	*Trichuris* sp*.*			
				*Trichostrongylus colubriformis*			
				*Teladorsagia circumcincta*			
				*Oesophagostomum columbianum*			
	Huancavelica	260	119 (45.8*)	*Lamanema chavezi*	Necropsy	Thesis	Gómez Escobar and Mallqui Saravia (2018)
	Puno	92	NR	*Strongylus* spp*.*	Rectal samples	Thesis	Quispe Pino (2019)
				*Nematodirus* spp*.*			
				*Lamanema* sp*.*			
				*Trichuris* sp*.*			
	Arequipa	288	NR	*Eimeria* spp*. (60.4)*	Rectal samples	Scientific research	Frezzato et al. (2020)
				*Eimeria macusaniensis (18.8)*			
				*Trichuris* spp*. (5.6)*			
				*Capillaria* spp*. (3.5)*			
				*Moniezia* spp*. (3.5)*			
				*Nematodirus/Marshallagia* spp*. (2.1)*			
				Strongylida *(1.4)*			
	Cuzco	78	68 (87.18)	*Eimeria lamae (85.90)*	Rectal samples	Scientific research	Gómez-Puerta et al. (2021)
				*Eimeria punoensis (62.82)*	Only *Eimeria* study		
				*Eimeria alpacae (53.85)*			
				*Eimeria macusaniensis (41.03)*			
				*Eimeria ivitaensis (5.13)*			
Ecuador	Imbabura	40	NR	*Eimeria* sp*. (67.50)*	Rectal samples	Thesis	Fierro Obregón (2010)
				*Trichostrongylus* sp*. (35.0)*			
				*Cooperia (32.5)*			
				*Marshallagia* sp*. (5.0)*			
				*Nematodirus* sp*. (12.50)*			
				*Trichuris* sp*. (12.50)*			
	Cotopaxi and Pichincha	406	NR	*Nematodirus* spp. *(45.5)*	Rectal simples	Scientific research	Salazar et al. (2014)
				*Bunostomum* spp. *(39.4)*			
				*Haemonchus* spp. *(27.5)*			
				*Cooperia* spp. *(14.5)*			
				*Ostertagia* spp. *(13.7)*			
				*Trichuris* spp. *(12.6)*			
				*Marshallagia* spp. *(6.1)*			
				*Strongyloides* spp. *(5.1)*			
				*Moniezia benedeni (5.9)*			
				*Moniezia expansa (4.4)*			
				*Eimeria lamae (18.2)*			
				*Eimeria macusaniensis (5.1)*			
	Pichicha	201	147 (73.0)	*Haemonchus* spp*. (77.9)*	Rectal samples	Thesis	Salazar Robayo (2015)
				*Nematodirus* spp*. (77.6)*			
				*Trichostrongylus* spp*. (77)*			
				*Bunostomum* spp*. (69.9)*			
				*Cooperia* spp*. (55.8)*			
				*Ostertagia* spp*. (50.4)*			
				*Oesophagostomum* spp*.(45.1)*			
				*Capillaria* spp*. (34.5)*			
				*Trichuris* spp*. (29.2)*			
				*Marshallagia* spp*. (25.6)*			
				*Lamanema* spp*. (22.1)*			
				*Strongyloides* spp*. (18.6)*			
				*Strongylus* spp*. (0.9)*			
				*Eimeria* spp*. (70.7)*			
				*Eimeria macusaniensis (29.3)*			
				*Moniezia expansa (19.4)*			
				*Moniezia benedeni (80.6)*			
	Cotopaxi	114	114 (100.0)	*Marshallagia* spp. *(9.6)*	Rectal samples	Thesis	Condor Tapia (2015)
				*Nematodirus* spp. *(42.1)*			
				*Strongylus* spp. *(14.9)*			
				*Trichostrongylus* spp. *(28.9)*			
				*Haemonchus* spp. *(13.2)*			
				*Ostertagia* spp. *(8.8)*			
				*Oesophagostomum* spp. *(9.6)*			
				*Bunostomum* spp. *(0.9)*			
				*Trichuris* spp. *(23.7)*			
				*Cooperia* spp. *(10.5)*			
				*Toxocara* spp. *(13.2)*			
				*Capillaria* spp. *(7.9)*			
	Cotopaxi	204	(71.0)	*Nematodirus* spp. *(89.0)*	Rectal samples	Thesis	Regalado Valdivieso (2015)
				*Bunostomum* spp. *(78.0)*			
				*Haemonchus* spp. *(43.0)*			
				*Capillaria* spp. *(31.0)*			
				*Trichostrongylus* spp. *(31.0)*			
				*Oesophagostomum* spp. *(28.0)*			
				*Lamanema chavezi (27.0)*			
				*Trichuris* spp. *(27.0)*			
				*Ostertagia* spp. *(26.0)*			
				*Cooperia* spp. *(20.0)*			
				*Marshallagia* spp. *(20.0)*			
				*Strongyloides* spp. *(10.0)*			
				Strongylida *(2.0)*			
				*Eimeria* spp. *(81.0)*			
				*Eimeria macusaniensis (25.0)*			
				*Moniezia benedeni (61.0)*			
				*Moniezia expansa (41.0)*			
	Cotopaxi	80	NR	*Ostertagia* sp. *(29.37)*	Rectal samples	Thesis	Panchi Lema (2021)
				*Nematodirus* sp*. (24.56)*			
				*Trichostrongylus* sp*. (5.79)*			
				*Haemonchus* sp*. (9.06)*			
				*Strongyloides* sp*. (18.89)*			
				*Trichuris tenuis (12.99)*			
				Coccidia *(83.75)*

**Table 2:** Gastrointestinal helminths and *Eimeria* spp. reported in llamas (*Lama glama*) across the entire nature distribution range. (* Calculated with published data, NR: no reported).

Country	Region	No. tested samples	No. positive (%)	Reported parasites (%)	Remarks	Type of publication	Reference
Argentina	Jujuy	15	15 (100.0)	*Fasciola hepatica*	Fecal samples	Scientific research	Cafrune et al. (1996a)
					Only *Fasciola* study		
	Jujuy	37	35 (95.0)*	*Trichuris tenuis*	Fecal samples and Necropsy	Scientific research	Cafrune et al. (1999)
					Only *Trichuris* study		
	Salta	2	2 (100.0)	Lamanema chavezi (100)*	Fecal samples and one Necropsy.	Scientific research	Cafrune et al. (2001)
				Trichuris tenuis (50.0)*	Farm llamas		
				*Trichostrongylus* spp. (50.0)*			
				*Cooperia* spp. (50.0)*			
				*Nematodirus* spp. (50.0)*			
	Jujuy	708	131 (18.5)	*Lamanema chavezi (13.9)*	Rectal samples	Scientific research	Cafrune et al. (2009a)
	Salta			*(31.7)*	Only *Lamanema* study		
	Catamarca			*(34.3)*			
	Jujuy	626	315 (50.3)	*Eimeria ivitaensis (0.4)*	Rectal samples	Scientific research	Cafrune et al. (2009b)
	Salta Catamarca			*(0.0)*	Only *Eimeria* study		
				*(2.0)*			
				*Eimeria macusaniensis (48.7)*			
				*(35.4)*			
				*(65.0)*			
	Jujuy	430		*Fasciola hepatica (21.6)*	Fecal samples	FAO project	Marin et al. (2009)
				*Lamanema chavezi (18.2)*			
				*Trichuris* sp*. (70.5)*			
				*Capillaria* sp*. (10.2)*			
				*Nematodirus* sp*. (1.1)*			
				*Strongyloides* sp*. (3.4)*			
				Strongylida *(5.7)*			
				Cestoda *(17.0)*			
				*Eimeria* spp*. (64.8)*			
				*Eimeria lamae*			
				*Eimeria alpacae*			
				*Eimeria punoensis*			
				*Eimeria ivitaensis*			
				*Eimeria macusaniensis*			
	Mendoza	2	2 (100.0)	*Fasciola hepatica (100.0)*	Fecal samples	Scientific research	Mera y Sierra et al. (2015)
				*Nematodirus* sp*. (50.0)*	Clinical signs of diarrhea		
	Salta	NR	NR	*Lamanema chavezi*	Dead llama with gastrointestinal symptoms.	Scientific research	Petrigh et al. (2019)
					DNA analysis		
	Catamarca	97	NR	Strongylida *(1.0)*	Rectal samples and necropsy	Thesis	Cardozo (2019)
		60		*(18.9)*	(+) indicates presence		
				*Trichuris* sp*. (15.50)*			
				*(23.3)*			
				*Toxocara* sp*. (72.30)*			
				*(1.6)*			
				*Lamanema chavezi (1.0)*			
				*(18.3)*			
				*Moniezia (+)*			
				*Strongyloides papillosus*			
				*(1.6)*			
				*Nematodirus* sp*. (0.0)*			
				*(11.6)*			
				*Camelostrongylus* sp*. (0.0)*			
				*(5.0)*			
				*Eimeria lamae (4.1)*			
				*(6.7)*			
				*Eimeria alpacae (7.2)*			
				*(26.7)*			
				*Eimeria punoensis (15.5)*			
				*(36.7)*			
				*Eimeria macusaniensis (10.3)*			
				*(28.3)*			
				*Eimeria ivitaensis (3.1)*			
				*(5.0)*			
				*Fasciola hepatica (3.15)*			
				*(2.6)*			
				*Ostertagia* sp*. (100.0)*			
				*+*			
				*Trichostrongylus* sp*. (8.0)*			
				*(15.0)*			
				*Cooperia* sp*. (0.0)*			
				*+*			
Chile	I Chile Region	150	NR	*Camelostrongylus mentulatus (73.3)*	Only abstract description	Scientific research	Alcaíno et al. (1991)
				*Trichostrongylus axei (11.3)*			
				*Ostertagia* sp. *(1.3)*			
				*Graphinema aucheniae (1.3)*			
				*Mazamastrongylus (Spiculopteragia) peruvianus (1.3)*			
				*Lamanema chavezi (61.3)*			
				*Nematodirus* sp. *(18.7)*			
				*Trichuris ovis (66.7)*			
				*Moniezia expansa (6.7)*			
	Araucanía, Temuco	45	NR	Strongylida	Fecal samples and field analysis	Scientific research	Müller (1998)
				*Nematodirus* sp.			
				*Ostertagia* sp.			
				*Nematodirus spathiger*			
				*Nematodirus filicolis*			
				*Trichostrongylus* sp*.*			
				*Cooperia* sp*.*			
	Los Ríos, Valdivia	32	(100.0)	*Capillaria* sp*.*	Rectal samples	Scientific meeting	Oyarzún-Ruiz et al. (2017)
				*Eimeria* sp.			
				*Eimeria macusaniensis*			
				*Fasciola hepatica*			
				*Moniezia* sp*.*			
				*Trichuris* sp*.*			
				*Nematodirus* sp*.*			
				Strongylida			
Bolivia	Oruro, Potosí, La Paz and Cochabamba	33	NR	*Lamanema chavezi (64.0)*	Fecal samples and necropsy	Scientific research	Spörndly and Nissen (2008) (in Mamani, 2012)
				*Nematodirus spathiger (55.0)*			
				*Nematodirus lamae (12.0)*			
				*Nematodirus abnormalis (15.0)*			
				*Camelostrongylus mentulatus (33.0)*			
				*Haemonchus contortus (15.0)*			
				*Trichuris* sp. *(42.0)*			
				*Graphinema aucheniae (12.0)*			
				*Marshallagia occidentalis (6.0)*			
				*Ostertagia ostertagi (12.0)*			
				*Cooperia oncophora (9.0)*			
				*Cooperia surnabada (3.0)*			
				*Trichostrongylus colubriformis (6.0)*			
				*Trichostrongylus vitrinus (3.0)*			
				*Trichostrongylus probolurus (6.0)*			
				*Skrjabinema* sp. *(3.0)*			
				*Moniezia* sp. *(3.0)*			
				*Fasciola hepatica (12.0)*			
				*Eimeria* spp. *(82.0)*			
Peru	Cuzco	NR	NR	*Eimeria lamae*	Rectal samples Mother and brood	Thesis	Mamani (2012)
				*Eimeria alpacae*			
				*Eimeria punoensis*			
				*Eimeria ivitaensis*			
				*Eimeria macusaniensis*			
				*Nematodirus spathiger*			
				*Nematodirus lamae*			
				*Lamanema chavezi*			
				*Trichuris* spp*.*			
				*Capillaria* spp*.*			
				Strongylida			
	Huancavelica	155	145 (93.55)	*Fasciola hepatica (9.7)*	Necropsy	Thesis	Fuentes Ríos (2013)
				*Haemonchus* sp. *(18.0)*			
				*Trichostrongylus axei (18.7)*			
				*Ostertagia* sp. (36.8)*			
				*Graphinema* sp. (15.5)*			
				*Camelostrongylus* sp. (11.0)*			
				*Nematodirus* sp. (83.22)*			
				Lamanema chavezi (45.2)*			
				*Cooperia* sp. (16.12)*			
				Trichostrongylus c. (15.5)*			
				*Bunostomum* sp. (6.45)*			
				*Moniezia* sp. (10.32)*			
				*Oesophagostomum* sp. (21.3)*			
				*Trichuris* sp. (78.7)*			
				*Skrjabinema* sp. (10.32)*			
	Junín, Jauja	97	(49.5)	*Fasciola hepatica*	Rectal samples	Scientific research	Flores et al. (2014)
					Only *Fasciola* study		
	Huancavelica	212	95 (44.8*)	*Lamanema chavezi*	Necropsy	Thesis	Gómez Escobar and Mallqui Saravia (2018)
Ecuador	Azuay, Sigsig	95	27 (28.4)	Strongylida	Fecal samples	Thesis	Zhiminaicela (2015)
				*Trichuris* sp.			
				*Bunostomum* sp.			
				*Nematodirus* sp.			
				Coccidia			
				*Trichostrongylus* sp*.*			
	Chimborazo, Millmahuanchi	44	NR	*Eimeria* sp. *(52.0)*	Rectal samples	Thesis	Gavilanes Loja (2016)
				*Strongyloides* sp. *(48.0)*			
				*Nematodirus* sp. *(14.0)*			
				*Trichostrongylus* sp. *(7.0)*			
				*Trichuris* sp. *(7.0)*			
				*Fasciola hepatica (9.0)*

**Table 3:** Gastrointestinal helminths and *Eimeria* spp. reported in vicuñas (*Vicugna vicugna*) across the entire distribution range. (* Calculated with published data, NR: no reported).

Country	Region	No. tested samples	No. positive (%)	Reported parasites (%)	Remarks	Type of publication	Reference
Argentina	Jujuy	187	30 (16.04)	*Fasciola hepatica*	Rectal samples	Scientific research	Cafrune et al., (1996b)
					Semi-captive		
					Only *Fasciola* study		
	Jujuy	69	45* (65.0)	*Trichuris tenuis*	Fecal samples	Scientific research	Cafrune et al. (1999)
					Semi-captive		
					Only *Trichuris* study		
	Jujuy	63	14 (22.2)	*Eimeria macusaniensis*	Rectal samples	Scientific research	Cafrune et al., (2009b)
	Salta	98	9 (9.2)		Semi-captive		
					Only *Eimeria* study		
	Jujuy	81 juveniles	81 (100.0)	*Eimeria punoensis (100)*	Rectal samples	Scientific research	Cafrune et al. (2014)
		154 adults	143 (92.8)	*(89.6)*	Captive		
				*Eimeria alpacae (85.1)*	Only *Eimeria* study		
				*(66.8)*			
				*Eimeria macusaniensis (82.7)*			
				*(15.5)*			
				*Eimeria lamae (48.1)*			
				*(27.2)*			
				*Eimeria ivitaensis (3.7)*			
				*(1.2)*			
	Jujuy	150	NR	Strongylida (40.66)	Rectal samples	Scientific research	Marcoppido et al. (2016)
				*Nematodirus* sp. (4.66)	Wild		
				Coccidia (7.33)			
				Cestoda (0.66)			
	Catamarca, Laguna Blanca	40	(2.5)	*Capillaria* sp.	Fecal samples	Thesis	Cardozo (2019)
				*Haemonchus* sp*.*			
				*Camelostrongylus* sp*.*			
				*Eimeria* spp*. (7.5)*			
				*Eimeria lamae*			
				*Eimeria alpacae*			
				*Eimeria punoensis*			
				*Moniezia* sp*.*			
				*Fasciola hepatica (12.5)*			
Bolivia	La Paz, Apolobamba	7 juveniles	7 (100.0)	Strongylida *(28.6)*	Rectal samples	Scientific research	Beltrán-Saavedra et al. (2011)
		25 adults	22 (88.0)	*(56.0)*	Wild		
				*Marshallagia* sp*.(71.4)*			
				*(32.0)*			
				*Lamanema* spp*. (42.9)*			
				*(16.0)*			
				*Nematodirus* spp*. (57.1)*			
				*(28.0)*			
				*Capillaria* sp*. (28.6)*			
				*(0.0)*			
				*Trichuris* sp*. (28.6)*			
				*(44.0)*			
				*Moniezia benedeni (14.3)*			
				*(0.0)*			
				*Eimeria punoensis (100.0)*			
				*(80.0)*			
				*Eimeria alpacae (100.0)*			
				*(88.0)*			
				*Eimeria lamae (42.9)*			
				*(12.0)*			
				*Eimeria macusaniensis (14.3)*			
				*(8.0)*			
	La Paz, Apolobamba	54 fecal samples	(100.0)	*Marshallagia* sp*.*	Fecal and dump samples	Scientific meeting	Condori et al. (2012)
		8 dump samples		*Nematodirus* sp*.*	Wild		
				*Trichuris* sp*.*			
				*Capillaria* sp*.*			
				*Lamanema chavezi*			
				*Moniezia benedeni*			
				*Moniezia expansa*			
				*Eimeria punoensis*			
				*Eimeria alpacae*			
				*Eimeria lamae*			
				*Eimeria macusaniensis*			
				*Cooperia oncophora*			
				*Cooperia macmasteri*			
				*Oesophagostomum columbianum*			
				*Ostertagia circumcincta*			
				*Trichostrongylus colubriformis*			
				*Trichostrongylus axei*			
				*Mazamastrongylus peruvianus*			
	Potosí, Tarija and Cochabamba	98	(73.5)	*Trichuris* spp. (32.7)*	Rectal samples	Thesis	Martela Mamani (2016)
				Lamanema chavezi (5.1)*	Semi-captive		
				*Marshallagia* spp. (4.1)*			
				Strongylida (30.61)*			
				*Capillaria* spp. (1.02)*			
				Moniezia benedeni (6.12)*			
				Fasciola hepatica (1.02)*			
				Eimeria punoensis (64.3)*			
				Eimeria alpacae (42.9)*			
				Eimeria peruviana (17.34)*			
				Eimeria lamae (11.22)*			
				Eimeria macusaniensis (1.02)*			
	La Paz and Oruro	84	(98.6)	*Marshallagia* spp. (32.14)*	Rectal samples	Thesis	Ruiz Hurtado (2016)
				*Lamanema* spp. (1.2)*	Semi-captive		
				Strongylida (38.1)*			
				*Nematodirus* spp. (10.7)*			
				*Trichuris* spp. (54.8)*			
				*Capillaria* spp. (1.2)*			
				Moniezia benedeni (3.6)*			
				Eimeria punoensis (39.3)*			
				Eimeria alpacae (40.5)*			
Peru	Cuzco	NR	NR	*Lamanema chavezi*	First report	Scientific research	Becklund (1963)
				*Nematodirus lamae*	Host: llama and vicuña		
					no access to original document		
	Ayacucho, Pampa Galeras	39	15 (41.0)	*Eimeria lamae*	NR	Scientific research	Bouts et al. (2003)(in Dubey, 2018)
				*Eimeria punoensis*			
	Tacna	120	(80.83)	*Trichuris* sp*. (81.44)*	Rectal samples	Thesis	Quispe García (2011)
				*Strongylus* sp*. (20.62)*	Semi-captive		
				*Nematodirus* sp*. (15.46)*			
				*Capillaria* sp*. (11.34)*			
				*Eimeria* sp*. (20.62)*			
	Huancavelica	80	(27.5)	*Fasciola hepatica*	Rectal and dump samples	Scientific research	Pizarro and Puray (2014)
					Only *Fasciola* study		
	Junín, Paccha	143	(32.9)	*Fasciola hepatica*	Rectal samples	Scientific research	Samamé et al. (2016)
					Wild		
					Only *Fasciola* study		
	Cajamarca	208	NR	Strongylida *(61.1)*	Rectal samples	Thesis	Curay Cabanillas (2018)
				*Nematodirus (39.4)*	Semi-captive		
				*Trichuris (26.9)*			
				*Capillaria (16.8)*			
				*Moniezia (8.7)*			
				*Cooperia (39.64)*			
				*Trichostrongylus (20.76)*			
				*Ostertagia (17.56)*			
				*Oesophagostomum (12.88)*			
				*Haemonchus (5.45)*			
				*Bunostomum (3.99)*			
	Cuzco	147	High	*Fasciola hepatica (2.0)*	Rectal samples	Scientific research	Angulo-Tisoc et al. (2021)
			NR	Strongylida *(42.1)*	Wild and captive		
				*Nematodirus* sp*. (6.8)*			
				*Nematodirus spathiger (26.5)*			
				*Trichuris* sp*. (4.0)*			
				*Eimeria* spp*. (85.0)*			
				*Moniezia* spp*. (2.7)*			
	Cuzco	115	(84.4)	Strongylida *(54.8)*	Fecal samples	Scientific research	Arias-Pacheco et al. (2021)
				*Nematodirus lamae (11.3)*	Semi-captive		
				*Nematodirus spathiger (13.9)*			
				*Trichuris* spp*. (9.6)*			
				Capillarid *(2.6)*			
				*Lamanema chavezi (13.0)*			
				*Eimeria alpacae (23.5)*			
				*Eimeria macusaniensis (34.8)*			
				*Eimeria lamae (6.1)*			
				*Eimeria punoensis (38.3)*			
				*Trichostrongylus* spp*.*			
				*Haemonchus* spp*.*			
				*Cooperia* spp*.*			
				*Teladorsagia* spp*.*			
				*Oesophagostomum* spp*.*			
				*Bunostomum phlebotomum*			
				*Gaigeria pachyscelis*			
Ecuador	Bolívar, Tungurahua and Chimborazo	200	NR	*Eimeria* spp. (69.85)	Dump samples	Thesis	Chacaguasay Cepeda (2016)
				Helminthes (68.84)			
				Larvae

**Table 4:** Gastrointestinal helminths and *Eimeria* spp. reported in guanacos (*Lama guanicoe*) across the entire distribution range. (* Calculated with published data, NR: no reported).

Country	Region	No. tested samples	No. positive (%)	Parasites reported (%)	Remarks	Type of publication	Reference
Argentina	Río Negro	3	NR	*Skrjabinema* sp*.*	NR	Scientific research	Larrieu et al. (1982) (in González-Rivas et al., 2019)
				*Trichuris ovis.*			
				*Trichostrongylus* sp*.*			
				*Trichostrongylus vitrinus*			
				*Trichostrongylus axei*			
				*Ostertagia ostertagi*			
				*Nematodirus filicolis*			
				*Nematodirus battus*			
				*Nematodirus lanceolatus*			
				*Nematodirus spathiger*			
				*Cooperia oncophora*			
				*Cooperia macmasteri*			
				*Capillaria* sp*.*			
	Tierra del Fuego	58	NR	*Haemonchus* sp.	Fecal samples	Scientific research	Navone and Merino (1989)
				*Nematodirus* sp.			
				*Marshallagia* sp.			
				*Ostertagia* sp.			
				*Trichostrongylus* sp.			
				*Oesophagostomum* sp.			
				*Chabertia* sp.			
				*Cooperia* sp.			
				*Eimeria* sp.			
	Chubut	20	12 (60.0)*	*Strongyloides* sp. (5.0)*	Rectal samples	Scientific research	Karesh et al. (1998)
				*Nematodirus* sp. (30.0)*	Free-ranging		
				*Marshallagia* sp. (10.0)*	Two animals in poor body conditions		
				*Trichostrongylus* sp.(15.0)*			
				*Trichuris* sp. (25.0)*			
				*Dictyocaulus* sp.(5.0)*			
	Chubut	12	NR	*Eimeria* spp. (83.3)*	Feces from necropsied animals	Scientific research	Beldoménico et al. (2003)
				Eimeria macusaniensis (75.0)*	Animals dead by starvation		
				*Nematodirus* sp. (75.0)*	Wild		
				*Marshallagia* sp. (66.6)*			
				*Trichuris* sp. (8.33)*			
				*Dictyocaulus filaria*			
				*Trichuris tenuis*			
				*Moniezia expansa*			
	Mendoza and San Juan	35	NR	*Eimeria* sp.	Only access to abstract	Scientific research	Borghi et al. (2004) (in Dubey, 2018)
				*Eimeria macusaniensis*			
	Mendoza	70	1 (1.4)	*Fasciola hepatica*	Fecal samples	Scientific research	Issia et al. (2007)
					First report		
					Wild		
					Only *Fasciola* study		
	Neuquen, Río Negro and Chubut	NR	(84.2)	*Fasciola hepatica*	Rectal samples Semi-captive	Scientific research	Larroza and Olaechea (2008)
					Only *Fasciola* study		
	Salta	4	1 (25.0)	*Eimeria macusaniensis*	Dung samples Semi-captive	Scientific research	Cafrune et al. (2009a)
					Only *Eimeria* study		
	Salta	4	3 (75.0)	*Lamanema chavezi*	Dung samples Semi-captive	Scientific research	Cafrune et al. (2009b)
					Only *Lamanema* study		
	Mendoza	224	(0.5)	*Fasciola hepatica*	Fecal samples	Scientific research	Issia et al. (2009)
					Wild		
					Only *Fasciola* study		
	Neuquén, Chubut and Río Negro	622	NR	*Nematodirus spathiger*	Necropsied animals	Scientific research	Olaechea et al. (2011)
				*Nematodirus oriatianus*	Semi-captive		
				*Nematodirus filicolis*			
				*Nematodirus abnormalis*			
				*Ostertagia ostertagi*			
				*Ostertagia trifurcate*			
				*Cooperia oncophora*			
				*Trichostrongylus colubriformis*			
				*Trichuris* spp.			
				*Dictyocaulus* spp.			
				*Moniezia* spp.			
				*Eimeria macusaniensis*			
				*Fasciola hepatica*			
	Mendoza	75 rectal	NR	*Eimeria* sp.	Rectal and field samples	Scientific research	Moreno et al. (2013)
		600 field		*Eimeria macusaniensis*	Wild		
				*Nematodirus* sp.			
	Mendoza	756	638 (84.4)*	*Nematodirus* spp.	Rectal samples	Scientific research	Moreno et al. (2015)
				*Trichuris* sp.	Wild		
				*Capillaria* sp.			
				*Strongyloides* sp.			
				*Moniezia benedeni*			
				*Eimeria lamae*			
				*Eimeria alpacae*			
				*Eimeria punoensis*			
				*Eimeria macusaniensis*			
				*Eimeria ivitaensis*			
	Mendoza	4	1 (25.0)*	*Fasciola hepatica*	Fecal samples	Scientific research	Mera y Sierra et al. (2015)
				*Eimeria* spp.	Semi-captive		
					One dead with diarrhea		
	Santa Cruz	NR	NR	*Nematodirus spathiger*	Fecal samples	Scientific research	Petrigh and Fugassa (2014)
					Wild		
					DNA analysis		
					Only *Nematodirus* study		
	Santa Cruz	15	(77.0)	*Nematodirus* spp.*(53.3)*	Fecal samples	Thesis	Taglioretti (2015)
				*Dictyocaulus* sp.(6.7)*	Wild		
				Strongylida *(20.0)*			
	Chubut	NR	NR	*Lamanema chavezi*	Fecal samples	Scientific research	Petrigh et al. (2019)
					Wild		
					DNA analysis		
					Only *Lamanema* study		
	San Juan	72	NR	*Eimeria* spp*.*	Fecal samples	Scientific research	González-Rivas et al. (2019)
				*Eimeria macusaniensis*	Wild		
				*Eimeria ivitaensis*			
				*Nematodirus* sp*.*			
				*Trichuris* sp*.*			
	Santa Cruz	4	NR	Capillarid eggs	Dump samples	Scientific research	Velázquez et al. (2020)
					Wild		
					Multi proxy analysis		
	Santa Cruz	10	10 (100.0)	*Lamanema chavezi(100.0)*	Necropsied animals	Scientific research	Santana et al. (2020)
				*Nematodirus* spp*. (100.0)*	Wild		
				*Capillaria* spp*. (60.0)*			
				*Trichuris* spp*. (40.0)*			
				Coccidia *(60.0)*			
Chile	Magallanes	NR	NR	*Ostertagia* sp*.*	NR	Scientific research	Cunazza (1982) (in Navone and Merino, 1998)
				*Trichostrongylus* sp*.*			
				*Nematodirus* sp.			
				*Oesophagostomum* sp*.*			
				*Trichuris* sp*.*			
				*Eimeria* sp*.*			
	Magallanes	15	10 (66.7)	*Eimeria macusaniensis (40.0)*	Fecal samples Semi-captive	Scientific research	Correa et al. (2012)
				*Nematodirus* sp*. (46.7)*			
				Strongylida *(20.0)*			
Peru	Cuzco	NR	(72.0)	*Strongylus* spp. *(75.0)*	No access to original document	Scientific research	Hurtado et al. (1985)
				*Lamanema chavezi (64.0)*			
				*Trichuris ovis (22.0)*			
				*Nematodirus* spp. *(14.0)*			
				*Eimeria macusaniensis (28.0)*			
	Ayacucho	132	71 (53.8)	Strongylida (31.8)	Field samples	Scientific research	Castillo et al. (2008)
				*Eimeria punoensis (21.2)*	Wild		
				*Eimeria alpacae (13.6)*			
				*Eimeria lamae (4.5)*			
				*Eimeria macusaniensis (15.9)*			
				*Trichuris* sp. *(8.3)*			
				*Nematodirus* sp. *(1.5)*			
				*Trichostrongylus* sp.			
				*Cooperia* sp.			
				*Ostertagia* sp.			
				*Bunostomum* sp.			
				*Mazamastrongylus peruvianus*			
				*Graphinema aucheniae*

The gastrointestinal parasites of each of the four SAC species compiled are summarized in Table 5. At least 36 parasitic helminths were registered. Twenty two genera of the Phylum Nematoda have been reported among the four species of SAC. Seventeen genera belong to the Order Strongylida (including 28 taxa identified to the species level), one genus belong to the Order Ascaridida, one genus belong to the Order Oxyurida, one genus belong to the order Rhabditida and two genus belong to the Order Enoplida. Three genera of the Phylum Platyhelminthes were also reported. One of them belongs to Cestoda (with two identified species) and one species belong to Trematoda. Respects to *Eimeria* spp. (Apicomplexa), five species have been identified. The prevalence of the reported parasitic infestations in many cases was 100% (Table 1, Table 2, Table 3, Table 4). This review displays that there is no one species more prevalent than another, but rather that the prevalence varies in each of the studies.

**Table 5:** Review of gastrointestinal parasites of South American Camelids.

Parasite species	Alpacas	Llamas	Vicuñas	Guanacos
**NEMATODA**				
STRONGYLIDA/ANCYLOSTOMATIDAE				
*Bunostomum* sp.	+	+	+	+
*Bunostomum phlebotomum*			+	
STRONGYLIDA/MOLINEIDAE				
*Lamanema* sp.	+	+	+	+
*Lamanema chavezi*	+	+	+	+
*Nematodirus* spp.	+	+	+	+
*Nematodirus spathiger*	+	+	+	+
*Nematodirus filicolis*	+	+		+
*Nematodirus abnormalis*		+		+
*Nematodirus battus*				+
*Nematodirus lanceolatus*				+
*Nematodirus oriatianus*				+
*Nematodirus lamae*	+	+	+	
STRONGYLIDA/TRICHOSTRONGYLIDAE				
*Camelostrongylus* sp.	+	+	+	
*Camelostrongylus mentulatus*		+		
*Cooperia* sp*.*	+	+	+	+
*Cooperia oncophora*		+	+	+
*Cooperia surnabada*		+		
*Cooperia mcmasteri*			+	+
*Graphinema* sp.	+	+		
*Graphinema aucheniae*		+		+
*Haemonchus* sp.	+	+	+	+
*Haemonchus contortus*		+		
*Marshallagia* sp*.*	+	+	+	+
*Marshallagia occidentalis*		+		
*Mazamastrongylus (Spiculopteragia) peruvianus*	+	+	+	+
*Ostertagia* sp*.*	+	+	+	+
*Ostertagia ostertagi*	+	+		+
*Ostertagia circumcincta*			+	
*Ostertagia trifurcate*				+
*Teladorsagia* spp.			+	
*Telodorsagia circumcincta*	+			
*Trichostrongylus* sp*.*	+	+	+	+
*Trichostrongylus colubriformis*	+	+	+	+
*Trichostrongylus axei*		+	+	+
*Trichostrongylus vitrinus*		+		+
*Trichostrongylus probolurus*		+		
STRONGYLIDA/CHABERTIDAE				
*Chabertia* sp.	+			
*Oesophagostomum* sp.	+	+	+	+
*Oesophagostomum columbianum*	+		+	
STRONGYLIDA/DICTYOCAULIDAE				
*Dictyocaulus* sp.	+			+
*Dictyocaulus filaria*				+
*Gaigeria pachyscelis*			+	
STRONGYLIDA/STRONGYLIDAE				
*Strongylus* sp.	+		+	
ASCARIDIDA/ASCARIDIDAE				
*Toxocara* spp.	+	+		
OXYURIDA/OXYURIDAE				
*Skrjabinema* sp.		+		+
RHABDITIDA/STRONGYLOIDIDAE				
*Strongyloides* spp.	+	+		+
*Strongyloides papillosus*		+		
ENOPLIDA/TRICHURIDAE				
*Capillaria* sp*.*	+	+	+	+
*Trichuris* sp*.*	+	+	+	+
*Trichuris ovis*		+		+
*Trichuris tenuis*		+	+	+
**PLATYHELMINTHES**				
CESTODA				
*Moniezia* sp.	+	+	+	+
*Moniezia expansa*	+	+	+	+
*Moniezia benedeni*	+		+	+
TREMATODA				
*Fasciola hepatica*	+	+	+	+
**APICOMPLEXA**				
*Eimeria lamae*	+	+	+	+
*Eimeria alpacae*	+	+	+	+
*Eimeria punoensis*	+	+	+	+
*Eimeria macusaniensis*	+	+	+	+
*Eimeria ivitaensis*	+	+	+	+
	36	44	36	42

## 4 **Discussion**

The present work is the first scientific review that provides detailed information about gastrointestinal parasite diversity of SAC throughout the entire native distribution range, encompassing a large number of documents. The records summarized here comprise documents dating from 1963 to 2022, with an increase in the last 10 years, whenever a wide production of scientific publications and graduate and postgraduate theses were produced. This point can be explained by the growing interest in recent years for SAC conservation and, in the other hand, for the economic interest on SAC around the world. The recopilated documents are focused mainly in gastrointestinal parasites studies from fecal samples or from necropsied animals, by microscopic techniques mostly. This implies that in many occasions it is not possible to identify the species. Molecular studies on camelid parasites are scarce. Although 101 works were recopilated, results highlight that a large number of the documents summarized are not published in indexed journals and are not easily accessible to a wider audience.

The highest species richness of gastrointestinal parasites was found in southern Peru, western Bolivia and central Patagonia. This agrees with the regions with the highest population of SAC and with the regions where more studies have been carried out. The important population of Peru explain that this country produced a great number of the available knowledge. However, it is mainly focused on alpaca. Numerous studies considered that latitude is one of the main factors correlated to parasite diversity and richness. Parasite diversity is expected to decrease in high latitude areas as result of lack of intermediate hosts or high mortality rates due to harsh conditions in winter (Krasnov et al., 2004; Lindenfors et al., 2007; Bordes et al., 2010; Poulin and Leung, 2011). The data on parasitic richness of SAC summarized in this paper so far do not allow us to observe a decrease pattern in their distribution throughout their extensive distribution. However, the parasite diversity display in this review should be taken with caution as it is subject to the number and type of samples analyzed in each region. So far, all parasite genera appear to be represented throughout the distribution range.

Most wild and domestic ungulate species have few host-specific parasites, which make up less than half of the total number of nematode parasite species found in a given host; and mostly have generalist parasites (Walker and Morgan, 2014). Across vast areas, SAC coexist with domestic herbivores such as sheep, goat and cattle. Furthermore, domestic SAC coexist with human populations. This proximity facilitates the exchange of parasites between domestic and wild animals and humans. Walker and Morgan (2014) found that domestic camelids (llamas and alpacas) have a high liability index (the degree to which a host species is vulnerable to infection with generalist parasites), with a value of 0.77. This index is designed to range from −1 (entirely host-specific parasites) to 1 (entirely parasites shared with the other group). This result displays that llamas and alpacas have mostly generalist species. In the present study, 22 genera of nematodes were reported, with at least 33 species. Of all of them, only five are known as SAC-specific nematodes: *Trichuris tenuis*, *Graphinema aucheniae*, *Camelostrongylus mentulatus*, *Nematodirus lamae*, and *Lamanema chavezi*. Most of the registered parasites in this review are generalist parasites, and are shared with domestic ungulates and wildlife species, such as *Ostertagia* spp., *Haemonchus contortus*, *Trichostrongylus* spp., *Cooperia* spp., and *Oesophagostomum*. From a sanitary point of view, it would be important to know if host-specific parasites dominate the communities of their hosts and generalist parasites tend to occur at lower abundances, or vice versa (Zaffaroni et al., 2000). In this review, is clear that there is not enough data to compare the abundance of different nematode species within a host. Further studies of the contribution of shared parasite species to total parasite burden rather than only species richness would be a step toward understanding the impact that generalist parasites have on SAC. In the other hand, several studies have looked at nematodes of wild ungulates in relation to domestic species. In the present review, domestic SAC displayed to have the same genera of parasites than wild SAC.

SAC have also been described as hosts for parasites of zoonotic importance such as *Fasciola hepatica*. This trematode was found in wild and domestic camelids, from the north of its distribution to the north of Patagonia. It was generally assumed that entry of *F. hepatica* to America coincided with the first arrival of the Europeans and their associated livestock in the late 15th century. Throughout the 500 years since its introduction, the parasite gained new definitive hosts among native species. This trematode is now widespread in livestock and can be mapped across the whole South America and certain regions of North America. Nonetheless in Argentina, eggs of *F. hepatica* have been observed in deer and camelid coprolites dating back to 2300 years B.P., prior to the arrival of the European cattle in the 15th century (Beltrame et al., 2020; Tietze et al., 2021). This shows that the presence of *Fasciola* in camelids is not only due to its transmission by European cattle.

Cestodes found in SACs are ruminant-related anoplocephalid of the genus *Moniezia*, identified in the four camelids species in a wide variety of environments with records that go from the north of its distribution reaching as far as northern Patagonia. Parasites of *Moniezia expansa* were identify in all SACs species, while *Moniezia benedeni* were identify in vicuñas, alpacas and guanacos. In the case of llamas, findings of cestodes were scarce, and in general it was only possible to identify the genus. Recently, *Moniezia* eggs were also found in coprolites from the middle to late-Holocene from the Argentinian Puna, evidencing the presence of this genus in SAC prior the European cattle arrival (Tietze et al., 2021).

There are five common species of *Eimeria* in SAC: *E. lamae*, *E. alpacae*, *E. punoensis*, *E. macusaniensis* and *E. ivitaensis* (Dubey, 2018). All *Eimeria* spp. were recorded in wild and domestic camelids throughout its distribution range. The most prevalent *Eimeria* found in guanacos was *E. macusaniensis*, but in general the most prevalent in SAC was *E. punoensis* while the least prevalent was *E. ivitaensis* (Marin et al., 2009; Rodríguez et al., 2012; Cafrune et al., 2014; Moreno et al., 2015). Of the five *Eimeria* species registered in SAC, *E. macusaniensis* is considered the most pathogenic, clinical symptoms can develop even before oocysts are registered in the feces. The host specificity along with the characteristic morphology of its oocyst makes it an effective indicator when identifying the host in coprological studies (Dubey, 2018). The presence of *E. macusaniensis* is reported even in ancient samples from Argentina, Chile and Peru (Fugassa et al., 2008; Beltrame et al., 2010; Taglioretti et al., 2015; de Souza et al., 2018; Le Bailly et al., 2020; Tietze et al., 2021).

When studying the interactions between wildlife, livestock, and their parasites, it will be important to understand the historical context and patterns of contact and relatedness between the host species. A useful tool for this focus is the paleoparasitology. Paleoparasitological studies on SAC have shown the presence of diverse parasitic species in ancient times, which demonstrate the presence of some of them in prehistoric times, before the arrival of the European fauna, and others in more recent archaeological levels. Within the archaeological records were found *Dictyocaulus* sp., *Fasciola hepatica*, *Lamanema chavezi*, *Moniezia* sp., *Nematodirus spathiger*, *Strongyloides* sp., *Trichuris* sp. (e.g. Taglioretti et al., 2017; Petrigh et al., 2021; Tietze et al., 2021). If we are to make predictions of changes in host–parasite interactions due to, for example, climate or land use change, or to introduction of exotic species, it is necessary to have an exhaustive knowledge of the parasitic diversity of SAC throughout the time and throughout their entire distribution. Environmental changes can modify the epidemiological pattern of parasitic diseases, with impacts on the economy, public health, and/or wildlife conservation (Rhyan and Spraker, 2010). This work highlights the need for a greater number of works to know with more certainty the parasitic fauna of SAC in the past and present, in order to achieve predictions that allow proper management of camelids for their future conservation. Furthermore, concerted research efforts are needed to understand the biology, epidemiology, diagnosis and distribution of the parasitosis of SAC along the entire distribution range to guide conservation decisions.

## 5 Conclusions

In summary, this review presents the first compendium of studies of gastrointestinal parasites of SAC throughout the native range of distribution. This serves as a baseline for future studies focused on elucidating the role that parasites play on SAC and further epidemiological research. Clearly, a better understanding of the extent and impact of parasites on SAC, at both the individual and population levels, is needed. This shortfall in knowledge is concerning for SAC conservation.

## Declaration of competing interest

The authors declare that they have no known competing financial interests or personal relationships that could have appeared to influence the work reported in this paper.

## References

[bib1] Aguirre D.H., Cafrune M.M. (2007).

[bib2] Alcaino H., Gorman T., Burgos M. (1991). Helmintiasis gastrointestinal en llamas (*Lama glama*) de la I Región de Chile. Parasitol. día..

[bib3] Angulo J., Tantaleán-Vidaurre M., Watanabe-Watanabe R., Del Solar Velarde J.M. (2015). Redescripción de *Lamanema chavezi* por microscopía óptica y microscopía electrónica de barrido. Rev. Investig. Vet. Peru..

[bib4] Angulo-Tisoc J.M., Pacheco J.I., Vélez V., García W., Castelo H., Gomez-Puerta L.A. (2021). Situación actual de la sarna e infecciones parasitarias en vicuñas (*Vicugna vicugna*) de la Región Cusco. Perú. Rev. Investig. Vet. Peru..

[bib5] Arias-Pacheco C., Pezo D., Mathias L.A., Tebaldi J.H., Castelo-Oviedo H., Lux-Hoppe E.G. (2021). Parasitological status of vicuñas (*Vicugna vicugna*) from southeastern Peru and its relationship with fiber quality. Trop. Anim. Health Prod..

[bib6] Auris Bellido E., Santiago Cahuana B. (2013).

[bib7] Becklund W.W. (1963). *Lamanema chavezi* gen. n., sp. n. and *Nematodirus lamae* sp. n. (Nematoda: Trichostrongylidae) from the Alpaca, *Lama pacos*, and the Vicuña, *Vicugna vicugna*. Perú. J. Parasitol..

[bib8] Beldomenico P.M., Uhart M., Bono M.F., Marull C., Baldi R., Peralta J.L. (2003). Internal parasites of free-ranging guanacos from Patagonia. Vet. Parasitol..

[bib9] Beltrame M.O., Fugassa M.H., Sardella N.H. (2010). First paleoparasitological results from late Holocene in Patagonian coprolites. J. Parasitol..

[bib10] Beltrame M.O., Pruzzo C., Sanabria R., Mora M.S. (2020). First report of prehispanic *Fasciola hepatica* from South America revealed by ancient DNA. Parasitology.

[bib11] Beltrán-Saavedra L.F., Nallar-Gutiérrez R., Ayala G., Limachi J.M., Gonzales-Rojas J.L. (2011). Estudio sanitario de vicuñas en silvestría del Área Natural de Manejo Integrado Nacional Apolobamba, Bolivia. Ecol. Boliv..

[bib12] Beltrán-Saavedra L.F., González-Acuña D., Nallar-Gutiérrez R., Ticona-Challco H. (2014). Estudio coproparasitario y ectoparasitario en alpacas (*Vicugna pacos* Linnaeus, 1758) de Apolobamba, con nuevos registros de Phthiraptera (Insecta) e Ixodidae (Acari), La Paz-Bolivia. J. Selva Andina Anim. Sci..

[bib13] Bordes F., Morand S., Krasnov B.R., Poulin R. (2010). Parasite diversity and latitudinal gradients in terrestrial mammals. The biogeography of host-parasite interactions.

[bib14] Borghi E.D., Araoz C., Jofré C., Duarte A., Mera y Sierra R.L. (2004). Gastrointestinal parasites of guanacos (*Lama guanicoe*) of Midwest Argentina (Mendoza and san Juan). Biocell.

[bib15] Bouts T., Fox M.T., Scheres G., Chávez A. (2003).

[bib16] Cafrune M.M., Rebuffi G.E., Gaido A.B., Aguirre D.H. (1996). *Fasciola hepatica* in semi-captive vicunias (*Vicugna vicugna*) in North West Argentina. Vec. Rec.

[bib17] Cafrune M.M., Rebuffi G.E., Cabrera R.H., Aguirre D.H. (1996). *Fasciola hepatica* en llamas (*Lama glama*) de la Puna Argentina. Vet. Argent..

[bib18] Cafrune M.M., Aguirre D.H., Rickard L.G. (1999). Recovery of *Trichuris tenuis* Chandler, 1930, from camelids (*Lama glama* and *Vicugna vicugna*) in Argentina. J. Parasitol..

[bib19] Cafrune M.M., Aguirre D.H., Rickard L.G. (2001). First report of *Lamanema chavezi* (Nematoda: Trichostrongyloidea) in llamas (*Lama glama*) from Argentina. Vet. Parasitol..

[bib20] Cafrune M.M., Marín R.E., Rigalt F.A., Romero S.R., Aguirre D.H. (2009). *Lamanema chavezi* (Nematoda: Molineidae): epidemiological data of the infection in South American camelids of Northwest Argentina. Vet. Parasitol..

[bib21] Cafrune M.M., Marín R.E., Rigalt F.A., Romero S.R., Aguirre D.H. (2009). Prevalence of *Eimeria macusaniensis* and *Eimeria ivitaensis* in south American camelids of Northwest Argentina. Vet. Parasitol..

[bib22] Cafrune M.M., Romero S.R., Aguirre D.H. (2014). Prevalence and abundance of *Eimeria* spp. infection in captive vicuñas (*Vicugna vicugna*) from the Argentinean Andean altiplano. Small Rumin. Res..

[bib23] Camareno E., Chávez A., Pinedo R., Leyva V. (2016). Prevalencia de *Eimeria* spp en alpacas de dos comunidades del distrito de Macusani, Puno, Peru. Rev. Investig. Vet. Peru..

[bib24] Cardozo P.A. (2019).

[bib25] Castillo H., Chávez A., Hoces D., Casas E., Rosadio R., Wheeler J.C. (2008). Contribución al estudio del parasitismo gastrointestinal en guanacos (*Lama guanicoe cacsilensis*). Rev. Investig. Vet. Peru..

[bib26] Chacaguasay Cepeda B.M. (2016).

[bib27] Chirinos A. (2017).

[bib28] Ciprian A. (2007).

[bib29] Cóndor Tapia D.M. (2015).

[bib30] Condori W., Gutiérrez E., Mamani W., Guzmán J. (2012). VI Congreso Mundial de Camélidos Sudamericanos. Arica-Chile.

[bib31] Contreras N., Chávez A., Pinedo V., Leyva V., Suárez F. (2014). Helmintiasis en alpacas (*Vicugna pacos*) de dos comunidades de Macusani, Puno, durante la época seca. Rev. Investig. Vet. Peru..

[bib32] Cordero A., Huanca W., Díaz P., López C.M., Panadero R., Fernández G., Lago N., Morrondo P., Díez-Baños P. (2011). Proceedings of the XII Congreso Ibérico de Parasitología SOCEPA.

[bib33] Correa L., Zapata B., Soto-Gamboa M. (2012). Gastrointestinal and blood parasite determination in the guanaco (*Lama guanicoe*) under semi-captivity conditions. Trop. Anim. Health Prod..

[bib34] Cunazza C., Navone G.T., Merino M.L. (1982).

[bib35] Curay Cabanillas J.J. (2018).

[bib36] de Souza M.V., da Silva L.G.R., Silva-Pinto V., Mendez-Quiros P., de Miranda Chaves S.A., Iñiguez A.M. (2018). New paleoparasitological investigations from the pre-inca to hispanic contact period in northern Chile. Acta Trop..

[bib37] Díaz G., Chero A., Purdy S., Lenin Maturrano L., Rosadio R. (2015). VII Congreso Mundial en Camélidos Sudamericanos.

[bib38] Díaz P., Panadero R., López R., Cordero A., Pérez-Creo A., López C.M., Fernández G., Díez-Baños P., Morrondo P. (2016). Prevalence and risk factors associated to *Eimeria* spp. infection in unweaned alpacas (*Vicugna pacos*) from Southern Peru. Acta Parasitol..

[bib39] Dubey J.P. (2018). A review of coccidiosis in South American camelids. Parasitol. Res..

[bib40] Farfan E.J. (2014).

[bib41] Fierro Obregón M.F. (2010).

[bib42] Flores B., Pinedo R., Suárez F., Angelats R., Chávez A. (2014). Prevalencia de fasciolosis en llamas y alpacas en dos comunidades rurales de Jauja. Perú. Rev. Investig. Vet. Peru..

[bib43] Fowler M.E. (2010).

[bib44] Franklin W.L. (2011). Handbook of the Mammals of the World.

[bib45] Frezzato G., Stelletta C., Murillo C.E.P., Simonato G., Cassini R. (2020). Parasitological survey to address major risk factors threatening alpacas in Andean extensive farms (Arequipa, Peru). J. Vet. Med. Sci..

[bib46] Fuentes Ríos M.A. (2013).

[bib47] Fugassa M.H., Sardella N.H., Taglioretti V., Reinhard K.J., Araujo A. (2008). Eimeriid oocysts from archaeological samples in Patagonia, Argentina. J. Parasitol..

[bib48] Gavilanez Loja M.J. (2016).

[bib49] Gomez Escobar G., Mallqui Saravia D. (2018).

[bib50] Gomez-Puerta L.A., Carrasco J., Robles K., Vargas-Calla A., Cribillero N.G., Arroyo G., Castillo H., Lopez-Urbina M.T., Gonzalez A.E. (2021). Coccidiosis in clinically asymptomatic alpaca (*Vicugna pacos*) crias from the Peruvian Andes. Parasitol. Int..

[bib51] Gonzalez-Rivas C.J., Borghi C.E., De Lamo D.A. (2019). Endoparásitos en guanaco (*Lama guanicoe*): Revisión de situación en Argentina y registros de la provincia de San Juan. Rev. Investig. Vet. Peru..

[bib52] Guerrero C.A. (1967). Coccidia (Protozoa: Eimeriidae) of the alpaca *Lama pacos*. J. Protozool..

[bib53] Guerrero C.A., Hernandez J., Bazalar H., Alva J. (1971). *Eimeria macusaniensis* n. sp. (Protozoa: Eimeriidae) of the alpaca *Lama pacos*. J. Protozool..

[bib54] Guerrero Díaz C., Navone G.T., Merino M.L. (1970).

[bib55] Hurtado E., Bustinza J., Sánchez C. (1985). *5.* Convención Internacional sobre Camélidos Sudamericanos 16-21 Jun 1985 Cuzco (Perú) (No. RISPAL No. 0295).

[bib56] Issia L., Ovejero R., Carmanchahi P., Pietrokovsky S., Wisnivesky-Colli C. (2007). V Congreso Latinoam Especialistas en Pequeños Rumiantes y CSA.

[bib57] Issia L., Pietrokovsky S., Sousa-Figueiredo J., Stothard J.R., Wisnivesky-Colli C. (2009). *Fasciola hepatica* infections in livestock flock, guanacos and coypus in two wildlife reserves in Argentina. Vet. Parasitol..

[bib58] Karesh W.B., Uhart M.M., Dierenfeld E.S., Braselton W.E., Torres A., House C., Puche H., Cook R.A. (1998). Health evaluation of free-ranging guanaco (*Lama guanicoe*). J. Zoo Wildl. Med..

[bib59] Krasnov B.R., Shenbrot G.I., Khokhlova I.S., Degen A.A. (2004). Flea species richness and parameters of host body, host geography and host ‘milieu. J. Anim. Ecol..

[bib60] Larrieu E., Bigatti R., Lukovich R., Eddi C.S., Bonazzi E.F., Gómez E., Niec R., Oporto N.R. (1982). Contribución al estudio del parasitismo gastrointestinal en guanacos (*Lama guanicoe*) y llamas (*Lama glama*). Gac. Vet..

[bib61] Larroza M., Olaechea F. (2008). XVII Reunión Científico Técnica de la Asociación Argentina de Veterinarios de Laboratorios de Diagnóstico.

[bib62] Le Bailly M., Goepfert N., Prieto G., Verano J., Dufour B. (2020). Camelid gastrointestinal parasites from the Archaeological Site of Huanchaquito (Peru): first results. Environ. Archaeol..

[bib63] Leguia G. (1991). The epidemiology and economic impact of llama parasites. Parasitol. Today.

[bib64] Leguía G., Casas E. (1998). *Eimeria ivitaensis* (Protozoa: Eimeridae) en alpacas *Lama pacos*. Rev. Per. Parasitol..

[bib65] Li O., Leguía G., Espino A., Duménigo B., Díaz A., Otero O. (2005). Detección de anticuerpos y antígenos para el diagnóstico de *Fasciola hepatica* en alpacas naturalmente infectadas. Rev. Investig. Vet. Peru..

[bib66] Lindenfors P., Nunn C.L., Jones K.E., Cunningham A.A., Sechrest W., Gittleman J.L. (2007). Parasite species richness in carnivores: effects of host body mass, latitude, geographical range and population density. Global Ecol. Biogeogr..

[bib67] Lizana Hilario E. (2016).

[bib68] Lopez Mejía M.E. (2014).

[bib69] Lucas J.R., Morales S., Barrios M., Rodríguez J., Vásquez M., Lira B., Torres B., Casas E., Espinoza J. (2016). Patógenos involucrados en casos fatales de diarrea en crías de alpaca de la Sierra Central del Perú. Rev. Investig. Vet. Peru..

[bib70] Mamani J. (2012).

[bib71] Marin R.E., Rodriguez D., Parreño V. (2009).

[bib72] Marino T.A.C. (2011). Determinación de resistencia antihelmíntica frente a ivermectina de nematodos gastrointestinales en alpacas (*Vicugna pacos*) Puno-Perú. Abanico Vet..

[bib73] Marcoppido G., Schapiro J., Morici G., Arzamendia Y., Vilá B. (2016). Coproparasitological evaluation of nematodes and coccidia in a wild vicuña (*Vicugna vicugna*) population in the Argentinean Andean Altiplano. J. Camelid Sci..

[bib74] Martela Mamani W. (2016).

[bib75] Masson M., Gutiérrez G., Puicón V., Zárate D. (2016). Helmintiasis y eimeriosis gastrointestinal en alpacas criadas al pastoreo en dos granjas comunales de la región Pasco, Perú, y su relación con el peso y condición corporal. Rev. Investig. Vet. Peru..

[bib76] Mera y Sierra R., Cantero F., González M. (2015). *Fasciola hepatica* en guanacos y llamas en un establecimiento de Malargüe, provincia de Mendoza. Rev. Argentina Zoonosis Enf. Infec. Emerg..

[bib77] Miller G.R., Gill A.L. (1990). Zooarchaeology at Pirincay, a formative period site in highland Ecuador. J. Field Archaeol..

[bib78] Moreno P.G., Eberhardt M.A.T., Lamattina D., Previtali M.A., Beldomenico P.M. (2013). Intra-phylum and inter-phyla associations among gastrointestinal parasites in two wild mammal species. Parasitol. Res..

[bib79] Moreno P.G., Schroeder N.M., Taraborelli P.A., Gregorio P., Carmanchahi P.D., Beldomenico P.M. (2015). La comunidad de parásitos gastrointestinales de guanacos silvestres (*Lama guanicoe*) de la reserva provincial La Payunia, Mendoza, Argentina. Mastozool. Neotrop..

[bib80] Müller R. (1998). Estudio del parasitismo gastrointestinal en llamas (*Lama glama*), en un predio en la IX Región de Chile. Memoria Título Médico Veterinario. Valdivia, Chile. U. Austral de Chile. Fac. Cs. Veterinarias..

[bib81] Navone G.T., Merino M.L. (1989). Knowledge of the endoparasitic fauna of *Lama guanicoe* Müller, 1776, from the Mitre Peninsula, Tierra del Fuego, Argentina. Bol. Chil. Parasitol..

[bib82] Neyra V., Chavarry E., Espinoza J.R. (2002). Cysteine proteinases Fas1 and Fas2 are diagnostic markers for *Fasciola hepatica* infection in alpacas (*Lama pacos*). Vet. Parasitol..

[bib83] Olaechea F., Larroza M., Raffo F. (2011).

[bib84] Olivera D., Grant J.L. (2009). Puestos de altura de la Puna Argentina: zooarqueología de Real Grande 1 y 6 y Alero Tomayoc. Rev. Mus. Antropol..

[bib85] Oyarzún-Ruiz P., Barrientos V., Rodríguez R., Almonacid A., Barrientos O., Painean J., Ortiz C., Ratto M. (2017). XXIV Congreso Latinoamericano de Parasitología.

[bib86] Palacios C., Tabacchi L., Chavera A., López T., Santillán G., Pezo D., Perales R. (2004). Eimeriosis en crías de alpacas: estudio anátomo histopatológico. Rev. Investig. Vet. Peru..

[bib87] Palacios C., Perales R., Chavera A., López T. (2005). Caracterización anátomo-histopatológica de enteropatías causantes de mortalidad en crías de alpaca. Rev. Investig. Vet. Peru..

[bib88] Palacios C.A., Perales R.A., Chavera A.E., Lopez M.T., Braga W.U., Moro M. (2006). *Eimeria macusaniensis* and *Eimeria ivitaensis* co-infection in fatal cases of diarrhoea in young alpacas (*Lama pacos*) in Peru. Vet. Rec..

[bib89] Panchi Lema L.S. (2021).

[bib90] Paredes J.M., Condemayta Z.C., Charaja L.C. (2009). Causas de mortalidad de alpacas en tres principales centros de producción ubicados en puna seca y humeda del departamento de Puno. Rev. Electron. Vet..

[bib91] Pérez H., Chávez A., Pinedo R., Leyva V. (2014). Helmintiasis y eimeriasis en alpacas de dos comunidades de Cusco. Perú. Rev. Investig. Vet. Peru..

[bib92] Petrigh R.S., Fugassa M.H. (2014). Molecular identification of *Nematodirus spathiger* (Nematoda: Molineidae) in *Lama guanicoe* from patagonia, Argentina. Helminthologia.

[bib93] Petrigh R.S., Cafrune M.M., Fugassa M.H. (2019). First mitochondrial and nuclear DNA sequences of *Lamanema chavezi* (Nematoda: Molineidae): novel findings to improve its identification in feces from South American camelids. Parasitol. Int..

[bib94] Petrigh R.S., Velázquez N.J., Fugassa M.H., Burry L.S., Mondini M., Korstanje M.A. (2021). Herbivore coprolites from the south-central Andes. A multiproxy study at los Viscos archaeological site, Catamarca, Argentina. J. Archaeol. Sci..

[bib95] Puray N., Pizarro, R. del Pilar (2014).

[bib96] Poulin R., Leung T.L.F. (2011). Latitudinal gradient in the taxonomic composition of parasite communities. J. Helminthol..

[bib97] Puicón V.H. (2018).

[bib98] Quina Quina Y. (2015).

[bib99] Quispe García H.H. (2011).

[bib100] Quispe Pino K.M.R. (2019).

[bib101] Regalado Valdivieso M.C. (2015).

[bib102] Rhyan J.C., Spraker T.R. (2010). Emergence of diseases from wildlife reservoirs. Vet. Pathol..

[bib103] Rodríguez A., Casas E., Luna L., Zanabria V., Rosadio R. (2012). Eimeriosis en crías de alpacas: prevalencia y factores de riesgo. Rev. Investig. Vet. Peru..

[bib104] Roncal Narváez C.A. (2014).

[bib105] Rosadio R., Londoñe P., Pérez D., Castillo H., Véliz A., Llanco L., Yaya K., Maturrano L. (2010). *Eimeria macusaniensis* associated lesions in neonate alpacas dying from enterotoxemia. Vet. Parasitol..

[bib106] Rosadio R., Maturrano L., Pérez D., Luna L. (2012). El complejo entérico neonatal en alpacas andinas. Rev. Investig. Vet. Peru..

[bib107] Ruíz Hurtado C.R. (2016).

[bib108] Salazar Robayo C.I. (2015).

[bib109] Salazar C., Regalado C., Mena L.M., Galecio J.S. (2014). XIV Congreso Panamericano de Ciencias Veterinarias.

[bib110] Samamé L.M., Chávez A., Pinedo R. (2016). Fasciolosis en vicuñas (*Vicugna vicugna*) de la sierra central del Perú. Rev. Investig. Vet. Peru..

[bib111] Santana J.L., Martínez A., Soulés A., Milicevic F., Cafrune Wierna M.M., Larroza M.P., Robles C.A. (2020). Hepatitis parasitaria por *Lamanema chavezi* en guanacos (*Lama guanicoe*) faenados en la Provincia de Santa Cruz, Argentina. Soc. Med. Vet.

[bib112] Shamseer L., Moher D., Clarke M., Ghersi D., Liberati A., Petticrew M., Shekelle P., Stewart L.A. (2015). Preferred reporting items for systematic review and meta-analysis protocols (PRISMA-P) 2015: elaboration and explanation. BMJ.

[bib113] Spörndly E., Nissen A.M., Mamani J. (2008). Evaluación de la carga parasitaria y su interacción madre-cría, desde el nacimiento al destete, en alpacas (*Vicugna pacos*) y llamas (*Lama glama*) en Cicas la Raya, Cusco. Cusco [Thesis.

[bib114] Taglioretti V. (2015).

[bib115] Taglioretti V., Fugassa M.H., Sardella N.H. (2015). Parasitic diversity found in coprolites of camelids during the Holocene. Parasitol. Res..

[bib116] Taglioretti V., Fugassa M.H., Rindel D., Sardella N.H. (2017). New parasitological findings for pre-Hispanic camelids. Parasitology.

[bib117] Tietze E., Urquiza S.V., Beltrame M.O. (2021). Paleoparasitological study of Holocene South American camelids (ca. 8970–470 years 14C BP) from an archaeological site, southern puna of Argentina (Antofagasta de la Sierra, Catamarca). Holocene.

[bib118] Torres Huacani L. (2017).

[bib119] Ueno H., Arandia R., Morales G., Medina G. (1975). Fascioliasis of livestock and snail host for *Fasciola* in the Altiplano region of Bolivia. Natl. Inst. Anim. Health Q..

[bib120] Valenzuela G., Leiva M.P., Quintana I. (1998). Estudio epidemiológico de larvas de nemátodos gastrointestinales en praderas pastoreadas por alpacas (*Lama pacos*) en Valdivia, Chile. Arch. Med. Vet..

[bib121] Velázquez N.J., Petrigh R.S., Benvenuto M.L., Martínez Tosto C., Camiolo I., Palacio P.I., Fugassa M.H., Valenzuela L.O., Burry L.S. (2020). Diseño y evaluación de un protocolo de extracción múltiple de restos vegetales, silicofitolitos, polen, parásitos, isótopos estables y ADN de heces de *Lama guanicoe*. A. Arqueol. Etnol..

[bib122] Vilá B.L. (2012).

[bib123] Vilá B., Arzamendia Y. (2020). South American Camelids: their values and contributions to people. Sustain. Sci..

[bib124] Walker J.G., Morgan E.R. (2014). Generalists at the interface: nematode transmission between wild and domestic ungulates. Int. J. Parasitol. Parasites Wildl..

[bib125] Wheeler J.C., Chikhi L., Bruford M.W., Zeder M.A. (2006).

[bib126] Yacobaccio H.D. (2021). The domestication of South American camelids: a review. Anim. Front..

[bib127] Zaffaroni E., Manfredi M.T., Citterio C., Sala M., Piccolo G., Lanfranchi P. (2000). Host specificity of abomasal nematodes in free ranging alpine ruminants. Vet. Parasitol..

[bib128] Zamorano R., Fredes F., Fuentes R., Parraguez V.H., Raggi L.A. (2012). VI Congreso Mundial de Camélidos Sudamericanos Arica-Chile.

[bib129] Zhiminaicela P.V. (2015).

